# Experimental Evaluation and Thermodynamic Analysis of Magnetic Fe_3_O_4_@La-Zr-MOFs for Highly Efficient Fluoride and Phosphate Removal

**DOI:** 10.3390/nano15131043

**Published:** 2025-07-04

**Authors:** Ziyi Zhang, Xinyun Chen, Yongyi Yu, Wenbin Pan, Ruilai Liu, Jiangyan Song, Jiapeng Hu

**Affiliations:** 1 College of Environment and Safety Engineering, Fuzhou University, Fuzhou 350001, China; ziyizhang1646@163.com (Z.Z.); yuyongyi0606@163.com (Y.Y.); wenbinpan@fzu.edu.cn (W.P.); 2 Key Laboratory of Green Chemical Technology of Fujian Province University, Fujian Provincial Key Laboratory of Eco-Industrial Green Technology, Wuyi University, Wuyishan 354300, China; 15980085777@163.com; 3 College of Resources and Environment, Fujian Agriculture and Forestry University, Fuzhou 350001, China; 4 Research Center for Environmental Functional Materials, State Key Laboratory of Water Pollution Control and Green Resource Recycling, College of Environmental Science and Engineering, Tongji University, Shanghai 200092, China

**Keywords:** phosphate removal, defluoridation, metal–organic frameworks, adsorption

## Abstract

Phosphate and fluoride ions are common water pollutants whose presence and excessive discharge cause potential hazards to the environment and human health. MOF materials commonly used to remove phosphate and fluoride ions are usually in powder form, with low recovery during regeneration. Herein, to address these issues, Fe_3_O_4_@La-Zr-MOFs magnetic composites for phosphate and fluoride removal were fabricated by means of the hydrothermal method. The adsorption properties of the adsorbent were systematically assessed by means of adsorption experiments. The magnetic Fe_3_O_4_@La-Zr-MOFs exhibited a magnetic recovery efficiency of 93%, and they could maintain outstanding adsorption performance at a broad range of pH values and superior selectivity for phosphate and fluoride ions. The adsorption process conformed to the Langmuir isotherm and pseudo-second-order models, indicating that it was dominated by monomolecular chemisorption. Further characterization of the Fe_3_O_4_@La-Zr-MOFs before and after adsorption and kinetic thermodynamic investigation revealed that the elimination mechanism of phosphate and fluoride ions by Fe_3_O_4_@La-Zr-MOFs includes ion exchange, electrostatic interactions, and surface complexation. This study demonstrates that magnetic reusable Fe_3_O_4_@La-Zr-MOFs composites have great promise for phosphate and fluoride removal and recovery.

## 1. Introduction

Phosphorus and fluoride are necessary elements for human health, as well as important components in the manufacturing of fertilizers and various significant chemicals. However, due to their widespread application, the pollution of natural waters with phosphorus and fluoride has caused global concern. Fluoride is one of the plentiful trace elements in the Earth’s crust and is primarily concentrated in minerals such as fluorite, fluorapatite, black mica, cryolite, fluoride salts, and certain clays [1]. The release of fluoride into water resources due to natural processes, biological factors, and human activities has resulted in over 250 million individuals in different regions of the world being exposed to water that is contaminated with fluoride [2]. Elevated fluoride levels in drinking water beyond the WHO threshold of 1.5 mg L^−1^ can pose health risks to individuals. The persistent intake of water with elevated fluoride levels can lead to dental and skeletal fluorosis. Additionally, it can result in metabolic and functional impairments to the nervous system, endocrine glands, kidneys, liver, and other organs [3]. Phosphorus is predominantly found in nature as phosphate. The discharge of excess phosphate from natural sources, human activities, industrial expansion, detergents, and urban activities leads to serious water pollution, along with pernicious environmental problems such as eutrophication and algal blooms. The excessive growth of nutrients in rivers, lakes, or other bodies of water tends to stimulate the depletion of oxygen levels in the water, leading to the deterioration of natural water ecosystems [4]. In China, urban sewage emission requirements state that emissions must stay beneath 0.5 mg L^−1^, while in the U.S., there are strict requirements for discharge levels to remain from 0.1 to 0.5 mg L^−1^. Although phosphate wastewater standards currently vary between countries, increasing concerns about eutrophication will lead to much more stringent phosphate wastewater standards for future wastewater treatment plants, such as 0.1 mg L^−1^ or lower [5]. Therefore, developing advanced technologies for the removal of phosphates and fluorides is crucial to meet the progressively more stringent emission standards.

Existing methods for phosphate and fluoride removal include chemical precipitation [6], ion exchange [7], electro-flocculation [8], membrane separation [9], and adsorption [10]. Adsorption is regarded as an effective technique for fluoride and phosphate removal due to its rapid and effective elimination capabilities, low costs, ease of operation, and potential for reuse [11]. Although various materials, including biochar, clay, resins, and activated alumina, have been applied for dephosphorization and defluoridation, these traditional adsorbents are limited by their low adsorption capacity, poor selectivity, and a narrow pH range of applicability [12]. Therefore, it remains urgent to discover an adsorbent with features such as cost-effectiveness, a wide range of applicability, and excellent reusability.

A metal–organic framework (MOF) is a crystalline material with inorganic metal ions/clusters and organic ligands. Different compounds can be used to develop various types of MOFs [13]. An MOF is always used in gas purification, drug transport, oil–water separation, energy storage and sensing, adsorption removal, as these are applications that require specific properties such as a highly porous nature, large surface area, high crystallinity, and adjustable pore size [14,15,16]. In addition, the structure of MOFs can be designed to meet a variety of needs, as different microstructures and properties can be obtained, and these frameworks are seen as potential adsorbents for the removal of various pollutants from water [17]. By controlling the connectivity of metal nodes and the number of unsaturated ligand sites, the microstructure of MOFs can be precisely engineered and adjusted, and by reducing the number of ligand-organic linkers or extending their chain lengths, it is possible to increase the diversity of pore size distributions of pristine MOFs and enhance their adsorption performance [18]. However, most types of MOFs are structurally unstable in aqueous solution, and the removal of pollutants usually leads to undesirable effects. Researchers have studied the functionalization of MOFs, e.g., how appropriate metal doping (the introduction of La, Zr, Ce, Fe, or Al) can provide abundant active sites and mesopores, and how the doping of multiple metals and the synergistic interaction of multi-metallic MOFs provide opportunities to customize the material properties and enhance the selectivity and adsorption capacity of MOFs, which can effectively raise the adsorption performance [19]. Lanthanum (La) is an environmentally friendly rare earth element that is abundant in the earth’s crust and has a high affinity for phosphates and fluoride [20]. Zirconium-based MOFs are suitable for adsorption applications involving Hg^2+^, organoarsenic acids, phosphates, and other contaminants due to their low toxicity, high chemical stability to acids/bases, and zirconium’s high degree of oxygenophilicity [21]. Su et al. [22] investigated the solvothermal synthesis of La-MOF-808 complexes doped with different amounts to remove phosphorus and reported that the highest adsorption of 0.75 La-MOF-808 was 287.71 mg g^−1^, which is greater than most of the adsorbents reported in the literature. In addition, the adsorbent could be effectively regenerated after five cycles. Min et al. [23] synthesized La-MOFs (La-BDC) doped UiO-66 using a simple solvothermal reaction. The results showed that the adsorption sites of La-BDC doped UiO-66 material were increased and enhanced the adsorption of phosphate compared to La-BDC, and the maximum adsorption capacity could reach up to 113.59 mg g^−1^. However, in the majority of cases, fabricated MOFs typically exist in a powdered form, causing difficulties for recycling in terms of solid–liquid separation and limiting their practical utilization in engineering processes. Therefore, in order to solve the difficulties of solid–liquid separation of MOF, the doping of magnetic iron oxide in composite materials has attracted significant interest [24]. Due to its expansive specific surface area, strong paramagnetism, easy separability, and relatively small size, magnetic iron oxide has been extensively researched in the field of water treatment [25]. Duan et al. [26] prepared a magnetic Al-MOF@Fe_3_O_4_ adsorbent using the hydrothermal method. It was shown that the composite material retained a sorption capacity of 71.4 mg g^−1^ for fluoride ions after five cyclic regeneration tests and had an excellent magnetic separation effect, which was favorable for the recycling of materials. Xiong et al. [27] prepared an Fe_3_O_4_@La-MOF material. Their result indicated that the material could be easily recycled using an external magnet, and its properties were not affected after 12 cycles.

In this study, magnetic Fe_3_O_4_@La-Zr-MOFs composites were fabricated by means of a hydrothermal method, and their phosphate and fluoride removal properties and mechanisms were thoroughly investigated. Static adsorption experiments were conducted on the Fe_3_O_4_@La-Zr-MOFs to explore the impacts of coexisting ions, initial concentrations of solutions, as well as their pH, reaction time, and temperature on the adsorption effectiveness. The practical application value of this magnetic composite was also explored using cyclic regeneration and real wastewater experiments.

## 2. Materials and Methods

### 2.1. Material Synthesis

#### 2.1.1. Synthesis of La-Zr-MOF

**Figure 1:**
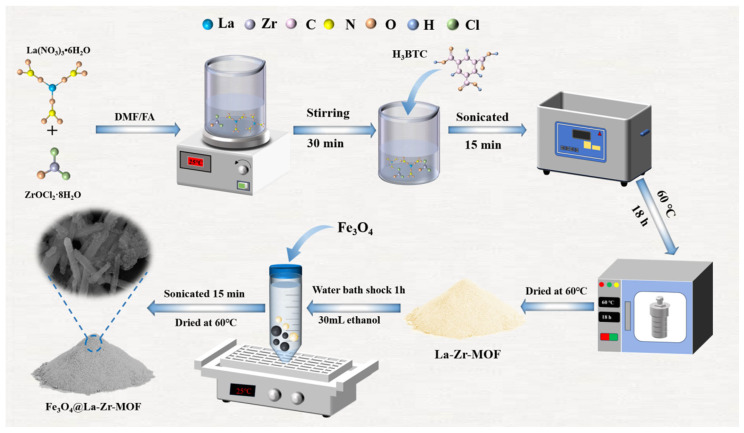
The synthesis process of Fe_3_O_4_@La-Zr-MOFs.

The preparation process of the Fe_3_O_4_@La-Zr-MOFs is shown in Figure 1. La(NO_3_)_3_·6H_2_O and ZrOCl_2_·8H_2_O were weighed into a beaker in a molar ratio of 1.25:1, supplemented with DMF/FA (31.5/31.5 mL), mixed and stirred for 30 min, supplemented with 0.2206 g of trimesic acid, ultrasonicated for 15 min, and then placed in a reactor at 60 °C for 18 h. The product was then centrifugally washed to neutrality with DMF and ethanol. The sample obtained after vacuum drying at 60 °C was the Zr-La-MOFs.

#### 2.1.2. Synthesis of Fe_3_O_4_@La-Zr-MOFs

A total of 0.1 g of the described above La-Zr-MOFs material was weighed, and Fe_3_O_4_ with a proportion of 2% was added to 100 mL centrifuge tubes. Next, 30 mL of anhydrous ethanol was added, and the mixture was oscillated in a water bath for 1 h, followed by ultrasonic oscillation for 15 min, static filtration, and drying under vacuum at 60 °C for 12 h. The resulting product was the Fe_3_O_4_@ La-Zr-MOFs.

### 2.2. Static Adsorption Experiments

NaF, KH_2_PO_4_, and distilled water were used to configure the fluoride and phosphate stock solutions for the experiment. The effects of the solution’s pH (2–10), initial F−concentrations (20, 30, 40, 50, 60, 80, and 100 mg L^−1^), initial phosphate concentrations (15, 25, 35, 45, 50, 55, and 60 mg L^−1^), co-existing anions (Cl^−^, NO_3_^−^, HCO_3_^−^ and SO_4_^2−^), reaction time (0–720 min), and oscillation temperature (25, 35, 45 °C) on fluoride and phosphate removal were studied. In the experiment, 0.01 g of adsorption material was placed into 50 mL of fluoride or phosphate solution, respectively, and shaken for 6 h. Then, we added 1 mL of the phosphate supernatant to a 50 mL volumetric flask, added 1 mL of ascorbic acid and 2 mL of ammonium molybdate, diluted the mixture to scale with distilled water, heated the water bath for 30 min, and measured the absorbance with a UV–visible spectrophotometer. The fluoride ion concentration was determined by means of the fluoride ion selective electrode technique in accordance with the national standard GB 7484-87.

## 3. Results and Discussion

### 3.1. Morphological Analysis

**Figure 2:**
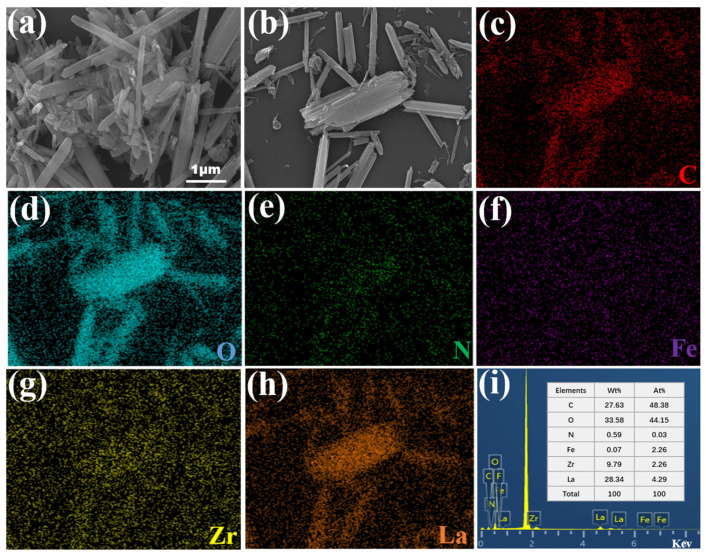
(**a**,**b**) SEM images, (**c**–**i**) mapping spectra (corresponds to (**b**)).

The topographic feature and surface elements of the Fe_3_O_4_@La-Zr-MOFs were analyzed employing SEM and EDS characterization. The findings are depicted in Figure 2a,b, demonstrating that the adsorbent possesses a unique rod-like structure, with irregular nanoparticles attached to the surface, and the size distribution varies between 0.1 and 50 μm. The rod-like structures usually have high aspect ratios; moreover, the attachment of irregular nanoparticles improves the density of active sites, increases the number of adsorption sites, and facilitates the interaction of pollutants with the surface of the material, thus improving the adsorption performance [28]. From the EDS analysis, as shown in Figure 2c–h, it can be seen that Fe_3_O_4_@La-Zr-MOFs consisted of the elements C, N, O, Fe, Zr, and La, and the mass percentages of Zr and La were 9.79% and 28.34%, respectively. The even dispersion of every element across the adsorbent surface facilitated the enhancement of phosphate and fluoride adsorption.

### 3.2. BET Analysis

**Figure 3:**
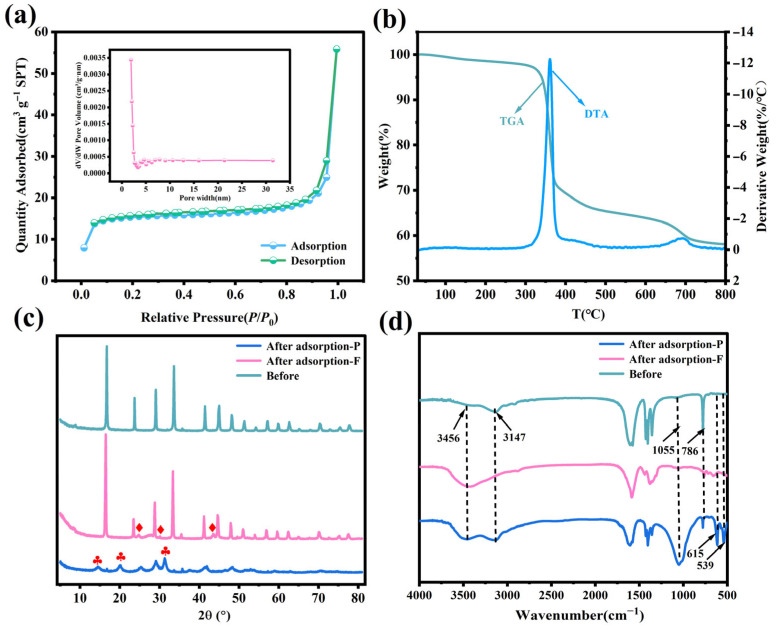
Fe_3_O_4_@La-Zr-MOFs characterization: (**a**) BET, (**b**) TGA, (**c**) XRD, (**d**) FTIR.

The N_2_ adsorption–desorption isotherms are displayed in Figure 3a. The BET specific surface area of the Fe_3_O_4_@La-Zr-MOFs is 68.28 m^2^ g^−1^, and their mean pore diameter is 5.0614 nm. The mesoporous structure, with pore diameters exceeding those of phosphate and fluoride ions, facilitates enhanced adsorption by allowing easier ion diffusion. Based on the IUPAC classification, the adsorption isotherm of the Fe_3_O_4_@La-Zr-MOFs was categorized as a type IV isotherm, signifying the presence of mesopores [29]. Figure 3a demonstrates that the adsorption capability improved rapidly at a relative pressure of *p/p*_0_ < 0.05, which was due to monolayer nitrogen adsorption on the material’s surface to facilitate the infiltration of condensed nitrogen into the pores [30]. The adsorption isotherm gradually ascended until reaching a saturation plateau when the relative pressure *p/p*_0_ within the range of 0.05 to 0.95. This behavior can likely be attributed to the gradual multilayer adsorption of nitrogen on the outer surface of the adsorbent. Beyond a relative pressure of *p/p*_0_ > 0.9, the mesopores and macropores interstices between particles gradually became saturated or filled, leading to a sharp surge in adsorption toward the maximum capacity [31].

### 3.3. Thermal Stability

The adsorbent materials were tested to assess their thermal stability at temperatures ranging from 30 to 800 °C. The TG and DTA curves are shown in Figure 3b. The TGA curves exhibit three distinct phases, with the initial phase showing a slight decrease in the interval below 350 °C, which is attributable to the decomposition of the physically or chemically adsorbed water and the loss of DMF solvent [32]. In the second stage, a weight loss of 33.91% can be observed in the interval from 350 to 480 °C due to the degradation of organic ligands. The mass reduction of about 6.94% in the third stage is owed to the structural decomposition of Fe_3_O_4_@La-Zr-MOFs. After 710 °C, the weight loss tends to stabilize. The above results suggest that Fe_3_O_4_@La-Zr-MOFs have good thermal stability and are suitable for practical applications.

### 3.4. XRD and FTIR

Figure 3c displays the XRD patterns of the Fe_3_O_4_@La-Zr-MOFs before and after adsorption. The obvious and sharp diffraction peaks that can be observed before the adsorption indicate that the material has a high degree of crystallinity. After fluoride removal, the main diffraction peaks did not shift or disappear, and the material’s crystal structure was not damaged, indicating good crystal stability. However, three new characteristic peaks appeared at 2θ = 24.83°, 29.91°, and 43.71°, indicating that ion exchange occurred between the adsorbent and fluoride ions, forming new crystals [33]. After the adsorption of phosphate, the crystal structure was disrupted, the intensity of the characteristic peaks was significantly reduced, some peaks were shifted, and new diffraction peaks appeared at 2θ = 14.47, 20.07° and 31.30°, demonstrating that interactions between the metal ions Zr and La and the phosphate occurred to form M-O-P inner-sphere complexes.

FTIR was used to obtain the functional group information of the adsorbed materials at 400–4000 cm^−1^ wavelengths. In Figure 3d, the strong broadband at 3600–3000 cm^−1^ belongs to the -OH of the physical adsorption of water [34]. The asymmetric stretching vibrational peaks of the carboxyl group (-COOH) appear at 1590 cm^−1^, while the peaks at 1434 cm^−1^ and 1406 cm^−1^ belong to the symmetric stretching vibration of -COOH [35]. In addition, the peaks at 786 and 661 cm^−1^ may be due to the bending vibrations of La-O and Zr-O, which indicates that the carboxylate of the organic ligand was successfully coordinated with the metal ions [32]. Upon contact with fluoride ions, the intensity of the -OH peak at 3456 cm^−1^ changed, proving the exchange between -OH and F^−^. The peaks associated with the metal groups diminished or vanished, proving the presence of electrostatic interactions between F^−^ and M-OH_2_^+^ [36]. Following phosphate adsorption, the intense and broad absorption peak at 1055 cm^−1^ likely corresponds to the v3 band vibration of HPO_4_^2−^ or H_2_PO_4_^−^, indicating that Fe_3_O_4_ @ La-Zr-MOFs and P formed La-O-P or Zr-O-P complexes [37,38]. Meanwhile, two new peaks appeared at 615 and 539 cm^−1^, associated with the bending vibration of O-P-O, and further proving the intra-sphere complexation occurring through ligand exchange [39].

### 3.5. VSM

**Figure 4:**
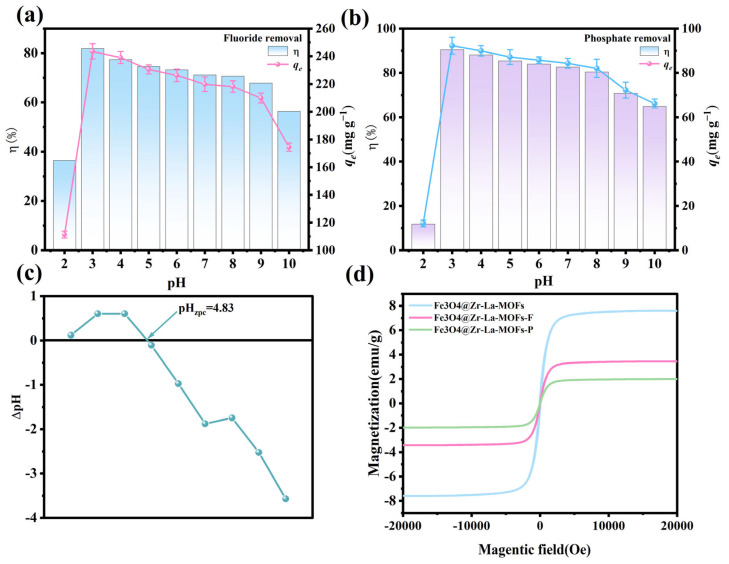
Effect of solution’s pH on (**a**) fluoride-removal, (**b**) phosphate-removal, and (**c**) zero-point charge; (**d**) magnetization curves.

The magnetic characteristics of the Fe_3_O_4_@La-Zr-MOFs were assessed using VSM both before and after the adsorption process. Figure 4d displays the magnetization of the Fe_3_O_4_@La-Zr-MOFs, showing that before and after the adsorption of fluoride and phosphates, the magnetization levels were 7.59, 3.43, and 1.98 emu/g, respectively. The magnetic hysteresis loop shows that the prepared magnetic Fe_3_O_4_@La-Zr-MOFs have some magnetism, and the saturation magnetization decreases after the adsorption of phosphate and fluoride, although this only has a tiny impact on the material’s recovery performance. Moreover, the absence of a noticeable hysteresis loop and anti-magnetism in the diagram suggests that the material has considerable paramagnetism. The magnetic test results further show that the magnetic performance of the adsorbent can be used to separate and recover the adsorbed material from the water.

### 3.6. Effect of Solution pH

Variations in pH led to changes in the surface charge of the adsorbent and the state of the contaminant anion, further impacting the affinity of the adsorbent towards pollutants [40]. Figure 4a demonstrates that fluoride removal is ineffective when pH = 2, because fluoride exists as HF in aqueous solution at lower pH levels, and its adsorption is poor [41]. The highest adsorption efficiency was achieved at pH = 3, while with increasing pH levels under strong alkaline conditions, the efficiency of the fluoride removal decreased slightly. This was due to the strong competition between fluoride ions and OH− under alkaline conditions, resulting in a decrease in adsorption capacity. Different pH levels in the solution affected the form of phosphate that was present: H_3_PO_4_ at pH < 2.12, mainly H_2_PO_4_^−^ at 2.12 < pH < 7.21, HPO_4_^2−^ at 7.21 < pH < 12.67, and mainly PO_4_^3−^ at pH > 12.67 [42]. According to Figure 4b, the removal efficiency of phosphate was 11.83% at pH = 2. This was because when phosphate mainly occurs in the form of H_3_PO_4_, it is difficult to adsorb by the adsorbent [43]. When pH = 3–10, the adsorption effect was improved; however, when pH > 8, the adsorption effect was weakened, and the removal rate decreased slightly. Figure 4c shows that the zero point charge (pH_zpc_) was 4.83. Below this value, the adsorbent surface became positively charged to facilitate adsorption, while above this value, the material surface was negatively charged because of protonation to discourage adsorption [44].

### 3.7. Effect of Initial Concentration

**Figure 5:**
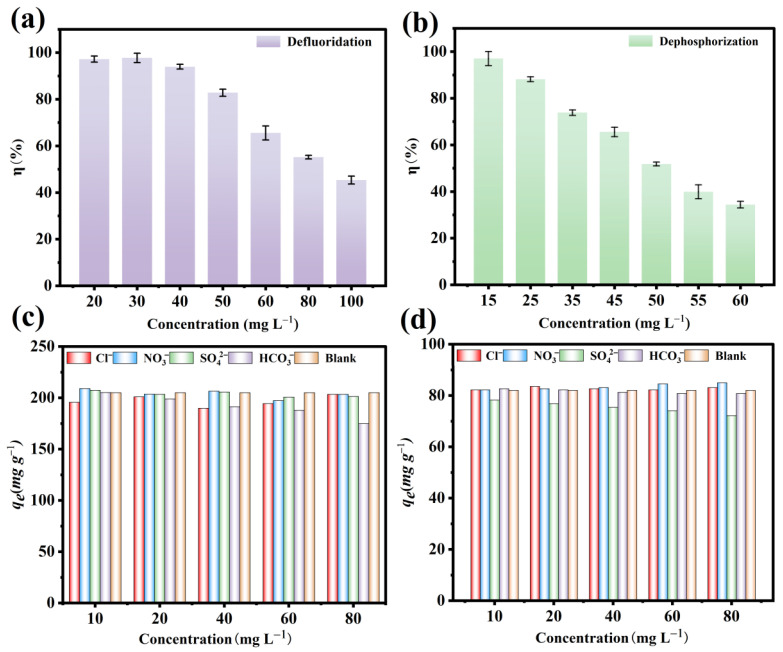
Effect of initial concentration on (**a**) fluoride removal and (**b**) phosphate removal; effect of co-existence on (**c**) fluoride removal and (**d**) phosphate removal.

Figure 5a,b illustrates that varying initial concentrations influenced the adsorption capabilities of the Fe_3_O_4_@La-Zr-MOFs. The removal efficiency was maintained above 97% for both phosphate and fluoride at concentrations lower than 15 mg L^−1^ and 20 mg L^−1^. This is due to the abundant adsorption sites on the surface of the Fe_3_O_4_@La-Zr-MOFs at low concentrations, which were sufficient for the adsorption of pollutants, resulting in a high adsorption efficiency. However, the removal efficiency decreased from 97.21% and 97.03% to 45.35% and 34.38% with increasing initial concentrations of fluoride and phosphate, respectively; these decreases occurred because the limited number of adsorption sites was not sufficient to adsorb large amounts of fluoride ions and phosphate under a fixed amount of Fe_3_O_4_@La-Zr-MOFs.

### 3.8. Effect of Coexisting Ions

In complex real water environments, we find not only one or two specific substances with toxic effects on humans that need to be removed, but rather a high number of potentially harmful substances or substances that interact with each other. The water body usually contains numerous other coexisting anions that interfere with the adsorption of phosphate and fluoride ions to a certain degree, as they will compete with phosphate and fluoride ions for active sites, thereby reducing the removal efficiency. Therefore, four anions (Cl^−^, NO_3_^−^, SO_4_^2−^, and HCO_3_^−^) were set up at different concentration gradients for the coexisting ion experiments. There were no significant effects of SO_4_^2−^ and NO_3_^−^ at increasing concentrations on fluoride ion adsorption (Figure 5c), which can be attributed to the weak binding ability of SO_4_^2−^ and NO_3_^−^ to the active sites on the adsorbent’s surface [45]. Nevertheless, HCO_3_^−^ inhibited fluoride ion adsorption, and when the concentration of HCO_3_^−^ ions was in the range of 10–80 mg L^−1^, the adsorbed amount of F^−^ decreased from 205 mg g^−1^ to 174 mg g^−1^. This indicated that the more coexisting HCO_3_^−^ there was, the more hydroxide ions were produced by means of hydrolysis, which increased the competition with fluoride ions, thus causing a reduction in adsorption. Figure 5d shows a slight promoting effect of NO_3_^−^ on phosphate adsorption, and the removal efficiency remained around 85% when the concentration of Cl^−^ and HCO_3_^−^ increased by 80 mg L^−1^. However, SO_4_^2−^ displayed a stronger inhibitory effect with increasing concentration. This was due to the close similarity in the ionic radius of sulfate (2.3 μm) and phosphate (2.38 μm), meaning that sulfate competes with phosphate by occupying the active sites, which weakens the electrostatic attraction of phosphate on the material surface, and therefore reduces the phosphate adsorption efficiency [4]. The above results indicate that the Fe_3_O_4_@La-Zr-MOFs have strong interference resistance and higher affinity for fluoride and phosphates than most coexisting interfering substances, indicating the practicality of eliminating target anions under intricate environmental conditions.

### 3.9. Adsorption Kinetics

**Figure 6:**
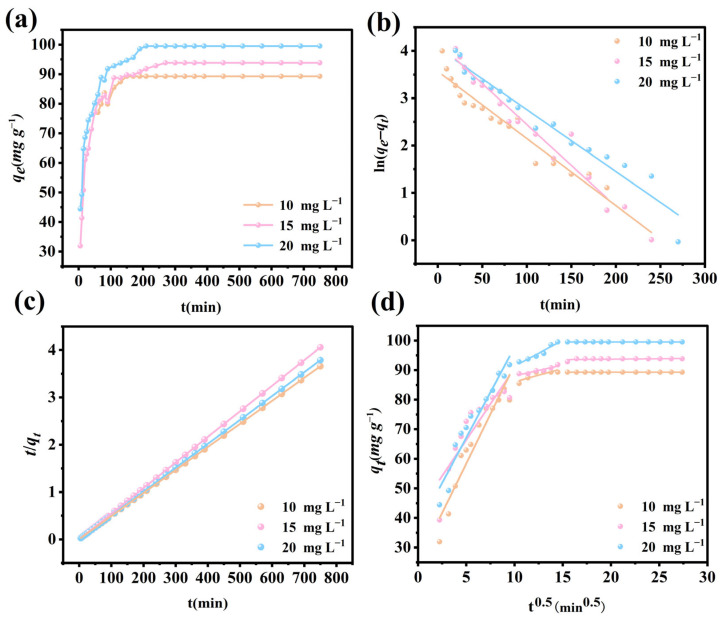
(**a**) Adsorption kinetic curves, (**b**) pseudo-first-order model, (**c**) pseudo-second-order model, and (**d**) intra-particle diffusion model for phosphate adsorption on Fe_3_O_4_@La-Zr-MOFs.

**Figure 7:**
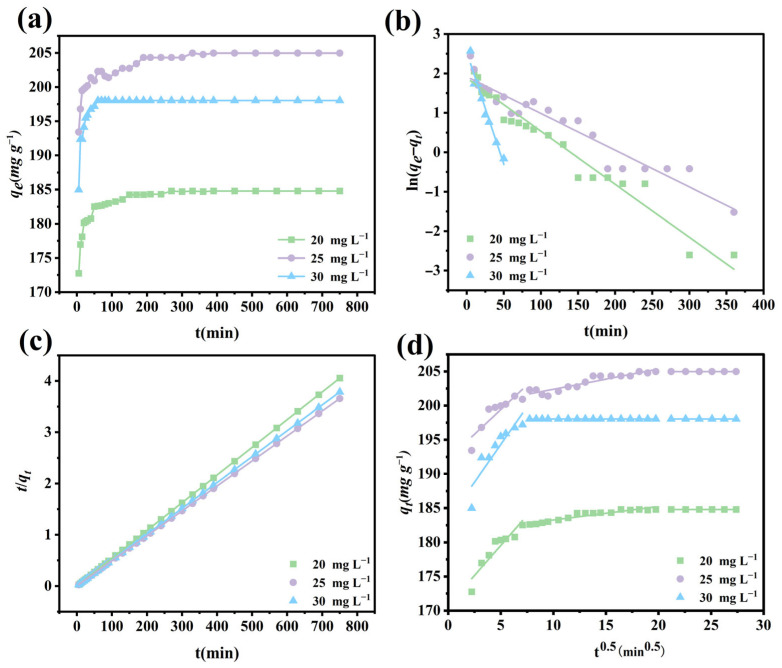
(**a**) Adsorption kinetic curves, (**b**) pseudo-first-order model, (**c**) pseudo-second-order model, and (**d**) intra-particle diffusion model for fluoride adsorption on Fe_3_O_4_@La-Zr-MOFs.

Adsorption kinetics experiments were carried out by altering the contact time to assess the reaction rate of the Fe_3_O_4_@La-Zr-MOFs for the adsorption of target anions. Figure 6a and Figure 7a show that the kinetic adsorption processes of the two pollutants are similar, with rapid adsorption within the first 60 min, a rapid increase with the increase in reaction time, and the adsorption equilibrium being reached after that point with the increasing saturation of the active sites on the material. The difference is that the adsorption time for fluoride ions is much shorter, with an adsorption equilibrium time of approximately 110 min. This is due to the relatively small size of fluoride, which requires a shorter distance to reach the adsorption sites on the Fe_3_O_4_@La-Zr-MOFs. Three adsorption kinetic models—pseudo-first-order, pseudo-second-order, and intra-particle diffusion were employed to fit and analyze the adsorption process, which are represented by Equations (S3)–(S5), respectively.

Figure 6 and Figure 7 present three different kinetic fitting curves, and the fitting parameters are listed in Table 1 and Table 2. The pseudo-second-order model fits of *q_e_* for phosphate and fluoride ions are closer to the experimental values and have higher correlation coefficients than those of the other two models, which demonstrates that the pseudo-second-order model describes the adsorption process with high accuracy and that the adsorption processes of both substances are chemisorption [46]. The model also shows that the adsorption rate is proportional to the square of the number of effective adsorption sites. This implies that the adsorption rate is not only affected by the number of available adsorption sites, but also closely related to the interactions between adsorption sites [47].

### 3.10. Adsorption Isotherms

Adsorption isotherm experiments can be used to predict the maximum sorption capacity for target pollutants, as well as to determine the interaction between the adsorption material and the pollutant ions [48]. Figure S4 depicts the relationship between the sorption capacity and temperature. As the temperature rose from 25 °C to 45 °C, the adsorbed amounts of both substances increased gradually, indicating that the adsorption process of phosphate and fluoride ions onto Fe_3_O_4_@La-Zr-MOFs is a heat-absorbing reaction. The experimental data were fitted and analyzed using the Langmuir and Freundlich models, and Equations (S6)–(S8) were used as the linear equations.

**Figure 8:**
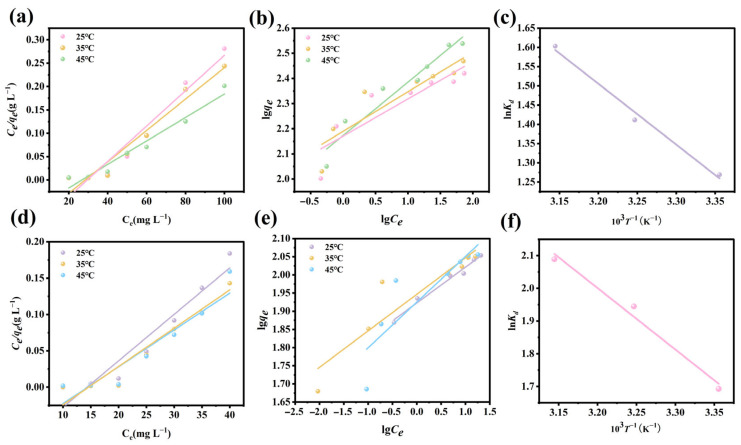
Langmuir model for (**a**) fluoride and (**d**) phosphate; Freundlich model for (**b**) fluoride and (**e**) phosphate; linear relationship between ln K_d_ and 10^3^T^−1^ for (**c**) fluoride and (**f**) phosphate.

The fitting outcomes of the two isotherm models are depicted in Figure 8, and the respective fitting parameters are detailed in Table S3. The Langmuir isotherm model exhibited an optimal fitting effect and higher R^2^ values, suggesting that the adsorption process of both pollutants mainly followed a monolayer adsorption process and formed a homogeneous adsorption layer [49]. The maximum Langmuir sorption capacities of the Fe_3_O_4_@La-Zr-MOFs for phosphate ions and fluoride ions were 197.23 mg g^−1^ and 398.4 mg g^−1^, respectively. Additionally, based on the adsorption equilibrium, the values of the parameters R_L_ were between 0 and 1, suggesting that the Fe_3_O_4_@La-Zr-MOFs have a strong affinity for both ions [50].

As illustrated in Table S5, the adsorption capacities of Fe_3_O_4_@La-Zr-MOFs and other comparable adsorbents have been studied. In comparison to the adsorbents that are documented in the existing literature, the synthesized Fe_3_O_4_@La-Zr-MOFs exhibited robust adsorption capabilities for phosphate and fluoride. These findings demonstrate that the doping of metal ions changes the coordination number of zirconium and lanthanum with ligands, resulting in flaws in the crystal lattice of MOFs, which is beneficial for exposing more Zr-OH and La-OH active sites for the adsorption of phosphate and fluoride, and significantly improves the adsorption capacity of MOFs [51].

### 3.11. Adsorption Mechanism

**Figure 9:**
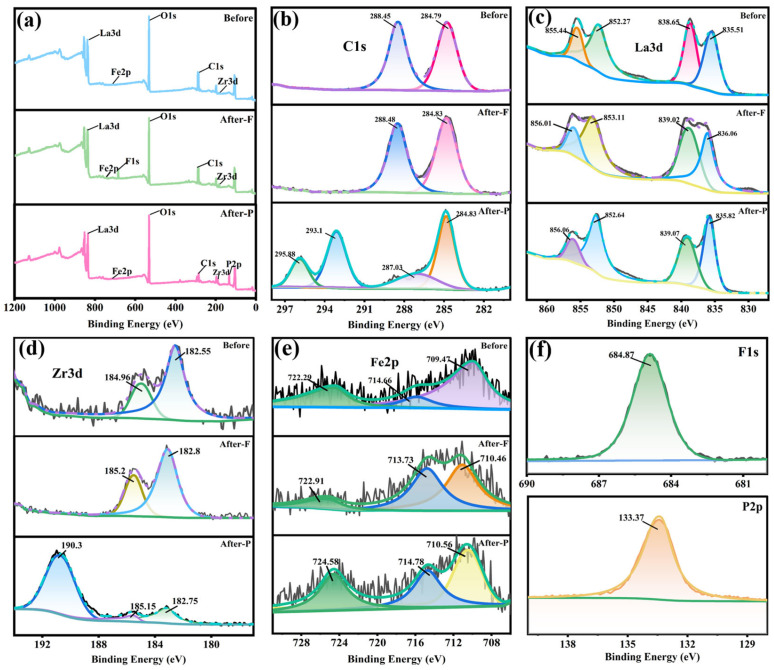
XPS spectra: (**a**) survey spectra, (**b**) C1s, (**c**) La3d, (**d**) Zr3d, (**e**) Fe2p, (**f**) F1s and P2p.

To more comprehensively investigate the adsorption mechanism of the Fe_3_O_4_@La-Zr-MOFs, XPS analysis was employed to explore the alteration in binding energy of elements following the adsorption of contaminants by the Fe_3_O_4_@La-Zr-MOFs. The main elements C, O, La, Zr, and Fe were identified in the survey spectra (Figure 9a), and the characteristic peaks of P and F were found to appear at 133.37 eV and 684.87 eV after the adsorption, which indicated the effective adsorption of phosphate and fluoride ions by the Fe_3_O_4_@La-Zr-MOFs. Compared with the standard P2p spectrum of KH_2_PO_4_ (134.0 eV), the P2p characteristic peak shifted to 133.27 eV after adsorption, and the decrease in P binding energy indicated that phosphate has a stronger affinity for Fe_3_O_4_@La-Zr-MOFs [52]. The F1s peak shifted to 684.87 eV compared with the standard characteristic peak of NaF, demonstrating the generation of La-F and Zr-F complexes. The XPS fine spectrum of C1s (Figure 9b) shows two peaks at 284.79 and 288.45 eV, which represent C-C and -O-C=O in the organic ligands, respectively [53]. After the adsorption of fluoride ions, the positions of the C-C and O-C=O peaks changed (by about 0.04 eV and 0.03 eV), and after the adsorption of phosphate, they shifted to 284.83 eV and 287.03 eV. The shifts in the binding energies of both sets of peaks suggested robust interactions or electron transfers between the fluoride ions, phosphate, and the adsorbent [33]. Among them, La3d_5/2_ and La3d_3/2_ each had two representative peaks (Figure 9c), which occurred at 835.51 eV and 838.65 eV and 852.27 eV and 855.44 eV, respectively. Upon the adsorption of fluoride, a shift in the peak positions towards higher binding energies occurred, suggesting that ionic exchange took place between F^−^ and the adsorbent. After phosphate adsorption, the binding energies of these two sets of peaks shifted to higher values (835.82 eV and 839.07 eV, and 852.64 eV and 855.06 eV), indicating that the electron transfer may have taken place in the valence band of La3d, forming La-O-P [54]. Before adsorption, the distinctive peaks of Zr3d_3/2_ and Zr3d_5/2_ were observed at 184.96 and 182.55 eV, respectively. After the adsorption of fluoride ions, a shift of approximately 0.3 eV in the peak positions occurred, which could be due to the formation of Zr substances [30]. After the adsorption of phosphate, a shift towards higher binding energies of 185.15 eV and 182.75 eV and a new characteristic peak at 190.3 eV occurred, which may be due to Zr-O-P inner-sphere complexes. this reduced the electron density of Zr, leading to higher binding energies [55]. The XPS spectra of Fe (Figure 9e) exhibit distinctive peaks at 722.29, 714.6, and 709.47 eV, corresponding to the Fe2p_1/2_ and Fe2p_3/2_ tracks, and the Fe^3+^ and Fe^2+^ signals appear at 714.6 eV and 709.47 eV, indicating the existence of Fe_3_O_4_ [56]. After adsorption, the peak area of Fe^2+^ in Fe_3_O_4_ decreased from 47.64% to 44.26% and 44.17%, while the content of Fe^3+^ increased from 16.61% to 37.86% and 29.73%, respectively. These results indicated that the Fe^2+^ underwent a reduction reaction during adsorption, and a large amount of Fe^2+^ in Fe_3_O_4_ was oxidized to Fe^3+^ [24].

Drawing upon previous research findings and the relevant characterization analyses, it is postulated that the adsorption process of fluoride ions and phosphates by Fe_3_O_4_@La-Zr-MOFs potentially features electrostatic interactions, ion exchange mechanisms, and complexation. In this study, the pH had a smaller effect on the adsorption of phosphate and fluoride ions, with a better performance at pH levels between 3 and 8. In acidic pH environments, the metal coordination hydroxyl group (M-OH) can protonate to (M-OH_2_^+^) for the adsorption of fluoride ions and phosphate, facilitated by electrostatically enhanced ion exchange reactions [57]. During surface complexation, phosphate and fluoride ions enter the surface of the adsorbent as ligands to displace the hydroxyl groups that are bound to La and Zr, thus generating complexes of complexes [58]. In our FT-IR analysis, after adsorption, the intense and broad absorption peak at 1055 cm^−1^ belongs to the typical P-O stretching vibration of the H_2_PO_4_^−^ or HPO_4_^2−^ groups, which suggests that the Fe_3_O_4_ @ La-Zr-MOFs formed La-O-P or Zr-O-P complexes with P through surface complexation. The enhanced intensity of the corresponding O-P-O stretching vibration peaks at 615 and 539 cm^−1^ suggests the existence of chemical coordination between La, Zr, and phosphates [20]. Following the adsorption of fluoride ions, alterations in the intensities of the -OH, -COO-, and M-O peaks were observed, suggesting the possible existence of electrostatic interactions between F^−^ and M-OH_2_^+^ and ion exchange reaction between F^−^ and OH^−^ on the Fe_3_O_4_@La-Zr-MOFs [59]. The XRD spectra (Figure 3c) showed that the crystallinity diminished after phosphate adsorption, which could be ascribed to the decrease in crystallinity caused by phosphate entering the lattice. At the same time, new crystals appeared, indicating that -OH is significant for phosphate elimination by Fe_3_O_4_@La-Zr-MOFs. After the adsorption of fluoride ions, three new diffraction peaks were detected in the Fe_3_O_4_@La-Zr-MOFs, indicating that the predominant removal mechanism in the solution involved ion exchange between the surface -OH and F^−^ to form new crystals [60]. In the XPS analysis, after the adsorption of phosphate and fluoride ions, the spectral peaks of La 3d and Zr 3d were shifted toward high binding energies, suggesting the formation of M-O-P complexes through metal-phosphate electron pair sharing, whereas the fluoride ions were electronically transferred between the metals Zr and La, generating Zr-F and La-F substances. The specific fluoride and phosphorus removal mechanism can be expressed by the following equation:

La/Zr-OH_2_^+^(s) + F^−^ → La/Zr-F(s) + H_2_O (1)

La/Zr-OH(s) + F^−^ → La/Zr-F(s) + OH^−^ (2)

La/Zr-OH(s) + H_2_PO_4_^−^/HPO_4_^2−^ → La/Zr-O-P + OH^−^ (3)

La/Zr-OH_2_^+^(s) + H_2_PO_4_^−^/HPO_4_^2−^ → La/Zr-O-P + H_2_O (4)

### 3.12. Regeneration Properties of Adsorbents and Application in Real Wastewaters

The reusability of materials in practical applications is an indispensable parameter when evaluating adsorbents. The experimental findings of the regeneration cycles are depicted in Figure S5. The results of the fluoride adsorption regeneration showed that after five regeneration tests, the removal efficiency of the Fe_3_O_4_@La-Zr-MOFs decreased to 70.65% and the sorption capacity was 147.53 mg g^−1^. This indicated that the Fe_3_O_4_@La-Zr-MOFs showed better recyclability for fluoride adsorption, whereas, for phosphate adsorption, the removal efficiency decreased to 42.50% after five cycles of regeneration. This decline in adsorption effectiveness may be linked to the exhaustion of active adsorption sites and the inadequate desorption of adsorbed contaminants from the saturated adsorbent. In summary, Fe_3_O_4_@La-Zr-MOF showed good recoverability for fluoride adsorption; however, there are some challenges for phosphate adsorption. To address the problem of decreased adsorption effect after phosphate regeneration, further research can be conducted to optimize the adsorbent structure, regeneration cycle method, or explore new regeneration techniques to improve its regeneration cycle performance and prolong the use of the adsorbent. The recovery rate was also studied in the experiment, and the results showed that the recovery rate could reach 93%, indicating that the magnetic adsorbent that was prepared in this study has a more satisfactory recovery effect.

We conducted practical application testing of the Fe_3_O_4_@La-Zr-MOFs using wastewater from an industrial park in Shaowu City. In the experiment, a polypropylene flask containing 50 mL of actual wastewater was spiked with 0.01 g Fe_3_O_4_@La-Zr-MOFs for 12 h at 25 °C in a shaker at 150 rpm. The parameter characteristics of the actual wastewater before and after treatment are shown in Table S6. The concentrations of fluoride ions and phosphates were 11.48 and 14.71 mg L^−1^ before adsorption, respectively. After treatment with Fe_3_O_4_@La-Zr-MOFs, the concentration decreased to 0.52 and 0.45 mg L^−1^, respectively. The fluoride ion concentration after adsorption complied with the maximum threshold concentration of 1.5 mg L^−1^ set by the WHO and the Chinese sanitary standard of 1.0 mg L^−1^, while the phosphate concentration reached the national emission level standard. In addition, the Fe_3_O_4_@La-Zr-MOFs also showed some ability to reduce the amount of COD in the solution, as well as NH_3_-N and other ions (e.g., SO_4_^2−^, HCO_3_^−^, and NO_3_^−^).

## 4. Conclusions

Herein, Fe_3_O_4_@La-Zr-MOFs were prepared to remove excessive fluoride and phosphate in aqueous solution. It was successfully proven that the Fe_3_O_4_@La-Zr-MOFs composites have superior adsorption abilities for phosphate and fluoride and a wider pH applicability range than previously reported adsorbents. The adsorption process of phosphate and fluoride ions by the Fe_3_O_4_@La-Zr-MOFs followed the Langmuir adsorption isotherm and the pseudo-second-order models, which indicates monolayer chemisorption. When pH = 3, the maximum Langmuir adsorption capacities for phosphate and fluoride ions were 197.23 mg g^−1^ and 398.4 mg g^−1^, respectively. The coexisting ion experiments showed that this absorbent has excellent selectivity and interference resistance. The phosphate and fluoride removal mechanisms included electrostatic action, ion exchange, and surface complexation. In addition, the VMS analyses indicated that the inclusion of magnetite (Fe_3_O_4_) enabled facile material separation using an external magnetic field, which facilitated recovery and reuse ability. Applied to actual wastewater, this material can reduce the concentration of fluoride and phosphate in industrial wastewater to meet national standards, showing its potential as a viable adsorbent for the elimination of phosphates and fluorides.

## Figures and Tables

**Table 1:** Kinetic parameters of Fe_3_O_4_@La-Zr-MOFs (phosphate removal).

Model	*C*_0_ (mg L^–1^)	*k*	*q_e_* (mg g^–1^)	R^2^
Pseudo-first order	10	0.0141	35.4864	0.9590
	15	0.0172	64.9105	0.9415
	20	0.0131	58.2800	0.9549
Pseudo-second order	10	0.0013	90.7441	0.9997
	15	0.0011	95.3288	0.9998
	20	0.0010	101.3171	0.9998

**Table 2:** Kinetic parameters of Fe_3_O_4_@La-Zr-MOFs (fluoride removal).

Model	*C*_0_ (mg L^–1^)	*k*	*q_e_* (mg g^–1^)	R^2^
Pseudo-first order	20	0.0134	6.5727	0.9514
	25	0.0093	6.8037	0.9241
	30	0.0568	12.6405	0.9608
Pseudo-second order	20	0.0076	185.1851	1
	25	0.0284	198.0198	1
	30	0.0027	205.3388	1

## Data Availability

The data are included within the article.

## Supplementary Materials

The following supporting information can be downloaded at: https://www.mdpi.com/article/10.3390/nano15131043/s1. References [61,62,63,64,65,66,67,68,69,70,71,72,73,74,75,76,77,78,79] are cited in the supplementary materials.
