# Supplementary material for: Experimental Evaluation and Thermodynamic Analysis of Magnetic Fe3O4@La-Zr-MOFs for Highly Efficient Fluoride and Phosphate Removal
Source: Nanomaterials (Basel). 2025 Jul 4;15(13):1043. doi: 10.3390/nano15131043 (PMC12250614; doi:10.3390/nano15131043)
Supplement: Supplementary file 1 [file nanomaterials-15-01043-s001.zip › nanomaterials-3710597-supplementary.pdf]

Supplementary Materials

Experimental evaluation and thermodynamic analysis of magnetic Fe_3O_4@La-Zr-MOFs for highly efficient fluoride and phosphate removal

Ziyi Zhang[1,2], Xinyun Chen[2,3], Yongyi Yu[1,2], Wenbin Pan[1], Ruilai Liu[2*], Jiangyan Song[4*], Jiapeng Hu[1,2*]

1. College of Environment and Safety Engineering, Fuzhou University, Fuzhou 350001, Fujian Province, China

2. Key Laboratory of Green Chemical Technology of Fujian Province University Fujian Provincial Key Laboratory of Eco-Industrial Green Technology, Wuyi University, Wuyishan 354300, Fujian Province, China

3. College of Resources and Environment, FUJIAN Agriculture and Forestry University, Fuzhou 350001, Fujian Province, China

4. Research Center for Environmental Functional Materials, State Key Laboratory of Water Pollution Control and Green Resource Recycling, College of Environmental Science and Engineering, Tongji University, Shanghai, 200092, P. R. China

* Corresponding authors: wyulrl@163.com (R. L.); sjya@tongji.edu.cn (J. Song); wyuwqhjp@163.com (J. Hu).

Text S1. Chemical reagents

Zirconium Oxychloride ($ZrOCl_2 \cdot 8H_2O$, 98% Adamas Reagent Co. Ltd.), Lanthanum Nitrate ($La(NO_3)_3 \cdot 6H_2O$, 99% Sinopharm Chemical Reagent Co. Ltd.), Benzene Tricarboxylic Acid (98%, Shanghai McLean Bio-Chemistry Co., Ltd.), AR-grade DMF, AR-grade Sodium Hydroxide (Sinopharm Chemical Reagent Co., Ltd.), Formic Acid (99%, Shanghai Aladdin Biochemical Technology Co., Ltd.), AR grade anhydrous ethanol, 36-38% hydrochloric acid (Sinopharm Chemical Reagent Co., Ltd.)

Text S2. Characterization

Tescan MIRA LMS scanning electron microscope (SEM), energy spectrum analyzer (EDS), and X-ray diffraction (XRD, Rigaku Smartlab SE) were used to study the micromorphology, surface elements, and crystal properties of Fe_3O_4@La-Zr-MOFs. The thermal stability of Fe_3O_4@La-Zr-MOFs were analyzed using TA Discovery TGA 550 thermogravimetric analyzer, and the specific surface area and pore size of the materials were evaluated using the Brunauer-Emmett-Teller (BET, Quantachrome AUTOSORB IQ). The functional group information and chemical composition of the adsorbents were determined by Fourier transform infrared spectroscopy (FTIR, Bruker-Vertex 70, Germany) and X-ray photoelectron spectroscopy (XPS, ESCALAB 250Xi).

Text S3. Adsorbent regeneration experiment

0.1 mol L^{-1} NaOH was used to rinse the saturated adsorbent to investigate the regeneration performance of Fe_3O_4@La-Zr-MOFs. Weigh 0.1 g of the sample, pour it into a 100 mL centrifuge tube, add 100 mL of phosphate and fluoride ion solutions of a certain concentration, respectively, Filter it and determine the concentration of the supernatant. The sample was collected after the reaction, poured into a 100 mL plastic centrifuge tube, added 100 mL of 0.1 mol L^{-1} NaOH solution, the samples were washed with deionized water until neutral and dried at 80 °C. the process was recorded as one cycle, and then followed the above steps for 5 cycles.

Text S4. Data analysis

The removal efficiency and adsorption capacity of fluoride and phosphate in the batch experiments were calculated by the following equations:

$$\eta = \frac{(C_0 - C_e)}{C_0} \times 100\% \qquad \text{(S1)}$$

$$q_e = \frac{V(C_0 - C_e)}{m} \qquad \text{(S2)}$$

Where, C_0 and C_e (mg L^{-1}) are the initial and equilibrium concentrations of the solution, respectively; V (mL) and m (g) denote the volume of the solution and the mass of Fe_3O_4@Zr-La-MOFs, respectively.

The kinetic parameters were fitted using the pseudo-first-order, pseudo-second-order and intra-particle diffusion models with the following expressions[61]:

$$\ln(q_e - q_t) = \ln q_e - k_1 t \qquad \text{(S3)}$$

$$\frac{t}{q_t} = \frac{1}{k_2 q_e^2} + \frac{t}{q_e} \qquad \text{(S4)}$$

$$q_t = k_p t^{\frac{1}{2}} + C \qquad \text{(S5)}$$

Where, q_t(mg g^{-1}) is the adsorbed amount of phosphate and fluoride ions corresponding to moment t, q_e is the adsorption capacity at adsorption equilibrium, and k_1 (($\min^{-1}$)and k_2 (g mg^{-1} $\min^{-1}$) are the adsorption rate constants for the pseudo-first-order and pseudo-second-order equations, respectively, k_p is the intra-particle diffusion rate constant (mg g^{-1} $\min^{-1/2}$); C is a constant.

The adsorption isotherm data were fitted using Langmuir and Freundlich models, the two models are shown in (6)-(7) respectively[62]:

$$\frac{C_e}{q_e} = \frac{1}{K_L q_m} + \frac{C_e}{q_m} \qquad \text{(S6)}$$

$$\lg q_e = \lg K_f + \frac{1}{n} \lg C_e \qquad \text{(S7)}$$

$$R_L = \frac{1}{1 + K_L C_0} \qquad \text{(S8)}$$

Where, q_m(mg g^{-1}) is the maximum adsorption capacity, q_e denotes the amount of fluoride or phosphate adsorbed at equilibrium, K_L is Langmuir's constant, K_f is Freundlich's constant, and the linear constant n characterizes the Freundlich adsorption model, reflecting the ease or difficulty of the adsorption; $n < 0.5$ indicates that the adsorption process is challenging, whereas $n = 2 \sim 10$ indicates a more favorable adsorption process. In addition, R_L is a separating factor indicative of adsorbability; values of $0 < R_L < 1$, indicate favorable adsorption, whereas $R_L > 1$ indicates an unfavorable process.

To further investigate the thermodynamic process of adsorption of phosphate and fluoride by the adsorbent, the entropy change ($\Delta S°$), enthalpy change ($\Delta H°$) and Gibbs free energy ($\Delta G°$) were analyzed and calculated by the following equations[63-64]:

$$K_d = \frac{q_e}{c_e} \tag{S9}$$

$$\Delta G^\circ = \Delta H^\circ - T\Delta S^\circ \tag{S10}$$

$$\ln K_d = -\frac{\Delta H^\circ}{RT} + \frac{\Delta S^\circ}{R} \tag{S11}$$

In these equations, $\Delta G°$ is the Gibbs free energy (kJ mol^{-1}), $\Delta S°$ is the entropy (J mol^{-1} K^{-1}), $\Delta H°$ is the enthalpy (kJ mol^{-1}), K_d is the equilibrium constant, R is the gas constant (8.314 J mol^{-1} K^{-1})

Text S5. Effect of Fe_3O_4 dosage

From **Fig S1** can be observe that when the proportion of Fe_3O_4 increases from 2 % to 5 %, the adsorption capacity of the adsorbent for F^- changes from 92.73 mg g^{-1} to 92.69 mg g^{-1}, and for phosphate changes from 65.79 mg g^{-1} to 64.37 mg g^{-1}. In this range, the adsorption capacity changes little. Considering the benefits, this experiment finally determines the Fe_3O_4@Zr-La-MOFs prepared when the proportion of magnetic raw materials is 2 % as the adsorbent for subsequent adsorption experiments.

Fig S2 shows the digital image of magnetic Fe_3O_4@La-Zr-MOFs prepared by the method solvent thermal method, the magnetic Fe_3O_4@La-Zr-MOFs are grey-black powdery, and the black color is mainly due to the Fe_3O_4 contained in the sample. The magnetic Fe_3O_4@La-Zr-MOFs were dispersed in an aqueous solution, and the magnetic adsorbent was clearly observed to be adsorbed by the magnet through a piece of ordinary magnet placed on the wall of the beaker.

Text S6. The optimal conditions for the synthesis of absorbent

In order to determine the optimal ratio of metal La to Zr, 0.3007 g of $ZrOCl_2 \cdot 8H_2O$ (1 mmol) was weighed, and different amounts of $La(NO_3)_3 \cdot 6H_2O$ were weighed according to the La to Zr molar ratios of 0.5:1, 0.75:1, 1:1, 1.25:1, 1.5:1, and added to a mixed solution of 63 mL DMF and formic acid (V/V =1:1), stirred for 30 min, then added 0.2206 g of H_3BTC and sonicated for 15 min to make it well mixed. The prepared solution was transferred to a reactor at 60 °C and reacted for 18 h. Then, it was centrifuged and washed three times, and the precipitate was finally dried under

vacuum at 80 °C for 12 h to obtain a white powder. The optimal metal ratio was screened by comparing the adsorption performance of the materials obtained from each ratio. The **Fig S3(a)** showed that when the molar ratio of La to Zr was 1.25:1, the adsorption of phosphate and fluoride ions by the material reached the maximum and the adsorption efficiency was the fastest.

After determining the optimal metal molar ratio, the next step was to investigate the optimal ratio of metal to organic ligand. Firstly, the total amount of metal was accurately weighed according to the molar ratios of 4:1,3:1,2:1,1:1,1:2, 1:3,1:4,1:5, and dissolved in a mixed solution of 63 mL DMF and formic acid (V/V =1:1). Subsequently, 0. 2206 g of H_3BTC was added and mixed. The same procedure as described above was followed. As can be seen from **Fig S3(b)**, the prepared material showed the highest removal of phosphate and fluoride ions and the best adsorption performance when the molar ratio of metal to H_3BTC was 2:1.

$La(NO_3)_3·6H_2O$ and $ZrOCl_2·8H_2O$ were weighed at a molar ratio of 1.25:1, dissolved in a mixed solution of DMF and formic acid (31.5 mL/31.5 mL), and mixed homogeneously for 30 min under magnetic stirring, then 0.2206 g of H_3BTC was added and ultrasonicated for 15 min, and the resultant mixed solution was transferred to a reactor for 18 h under constant temperature at 60 °C, 90 °C, 120 °C and 150 °C, respectively. The resulting mixed solution was transferred to the reactor and the reaction was carried out at constant temperature at 60 °C, 90 °C, 120 °C and 150 °C, respectively, for 18 h. 0.01 g of the Fe_3O_4@La-Zr-MOFs sample was injected into 20 mg L^{-1} phosphate and fluoride ions, and the absorbance was measured spectrophotometrically to calculate the removal efficiency and adsorption amount of phosphate and fluoride. The data in **Fig S3(c)** show that the Fe_3O_4@La-Zr-MOFs material prepared at 60 °C has the best adsorption performance and removal effect. This may be because that at higher temperatures (e.g., 120 °C and 150 °C), the by-products generated during the reaction tend to clog the pores, which prevents the good growth of crystals and ultimately reduces the adsorption capacity of the material.

To investigate the effect of reaction time on the adsorption efficiency, the reaction time was set at 6 h, 12 h, 18 h, 24 h and 30 h under the conditions of optimal reaction temperature and optimal molar ratio, respectively. The experimental steps were the same as those above, and it can be seen from **Fig S3(d)**, the best adsorption effect on phosphate and fluoride was achieved by the Fe_3O_4@La-Zr-MOFs prepared at 18 h. This may be because when the reaction time is too short, the crystal structure of the material is incomplete and the adsorption sites are insufficient, while too

long a reaction time may cause structural changes in the material or the generation of by-products, which reduces the adsorption performance.

Text S7. intra-particle diffusion model studies

The mechanism of adsorbate transfer and transport from the solid-liquid interface to the solid adsorbent is inferred from the intra-particle diffusion model, whose intercept provides us with the notion of boundary layer thickness, the larger the value of the intercept, the greater the contribution of surface adsorption in the rate-determining step[65]. By analyzing the fitted plots of intra-particle diffusion (**Fig 6(d)**, **Fig 7(d)**) and the fitted parameters (**Table S1, Table S2**), it can be observed that there are two adsorption processes for the adsorption of Fe_3O_4@La-Zr-MOFs. The first adsorption phase occurs rapidly during the first 30 min, which is due to the strong interaction between the outer surface of the adsorbent and the contaminant, and this phase is mainly controlled by membrane diffusion. The slow adsorption in the second stage is due to the diffusion of contaminants into the adsorbent-rich pores, where the diffusion resistance determines the reduction of the adsorption rate, and the second stage is mainly controlled by intra-particle diffusion[66]. The intercepts of the linear fits for both phases were not zero, implying that intra-particle diffusion was not the only rate-dominant mechanism controlling the adsorption of phosphate and fluoride ions by Fe_3O_4@La-Zr-MOFs[67].

Text S8 Thermodynamics studies

The effect of temperature on the adsorption of phosphate and fluoride ions was further explored using thermodynamic parameters including enthalpy change (ΔH^o), entropy change (ΔS^o), and Gibbs free energy change (ΔG^o), which can be calculated using equations (S8)-(S10).

The linear relationship between *lnK* and *1/T* is shown in **Fig 8(c)**、**Fig 8(f)**. The corresponding thermodynamic parameters are summarized in **Table S4**. The positive enthalpies of adsorption of phosphate and fluoride ions clearly indicate that the adsorption process is a heat-absorbing reaction, and the increase in temperature increases the diffusion rate of the pollutants in solution, which is favorable for more pollutants to adsorb with the adsorbent surface. Positive values of ΔS^o indicate that a series of reactions occurring on the surface of the adsorbent to increase the degree of mixing at the solid-liquid interface[68]. The negative values of ΔG^o at 298, 308 and 318 K indicate that the removal of phosphate and fluoride ions is spontaneous and favorable. Moreover, If the ΔH^o value is between 2.1 and 20.9 kJ mol^{-1} or ΔG^o is between −20 and 0 kJ mol^{-1}, it means that the process is

physisorption. If the ΔH^o value is in the range of 80-200 kJ mol^{-1} or ΔG^o is in the range of −400 to −80 kJ mol^{-1}, the process is chemisorption[69]. The experimentally calculated ΔH° and ΔG° are consistent with the range of physical adsorption, indicating that there is a physical adsorption mechanism in the adsorption process of Fe_3O_4@La-Zr-MOFs under this temperature change; moreover, ΔG^o gradually decreases with the continuous increase of temperature, which indicates that the effect of physical adsorption decreases with the increase of temperature. Therefore, the adsorption process of Fe_3O_4@La-Zr-MOFs on phosphates includes both chemical and physical adsorption and exhibits spontaneous heat absorption.

Text S9 Analysis of regeneration results of Fe_3O_4@La-Zr-MOFs

For phosphate adsorption, the removal decreased to 42.50% after five cycles of regeneration, and the decline in adsorption effectiveness may be linked to the exhaustion of active adsorption sites and inadequate desorption of adsorbed contaminants from the saturated adsorbent and discussed in the revision with the red color. The poor regeneration of phosphates compared to fluoride ions can be explained by FTIR and SEM plots. From the SEM image (Fig S6) we can see that after regeneration of fluoride ion adsorption, the structure of the material maintains a certain integrity, whereas after phosphate adsorption it becomes loose compared to the tightly clustered structure before adsorption, indicating that the material is damaged after adsorption regeneration. From the FTIR diagram (Fig S7), we can see that the intensity of some of the characteristic peaks weakened after fluoride ion adsorption, while some of the peaks changed after phosphate adsorption accompanied by the appearance of three new peaks. This indicates that some complexes were generated after the reaction between phosphate and adsorbent. Combined with the above analysis of SEM and FTIR diagrams we speculate that the poor regeneration effect of phosphate adsorption may be due to a series of reactions between the adsorbent and phosphate to damage the structure of the adsorbent, the active sites on the surface of the material is reduced, and these reactions produce complexes that are difficult to be desorbed, so that the binding capacity of the material with OH^- during regeneration becomes weaker and thus lead to the regeneration results are poor. To address the problem of decreased adsorption effect after phosphate regeneration, further research can be conducted to optimize the adsorbent structure, regeneration cycle method or explore new regeneration techniques to improve its regeneration cycle performance and prolong the use of adsorbent.

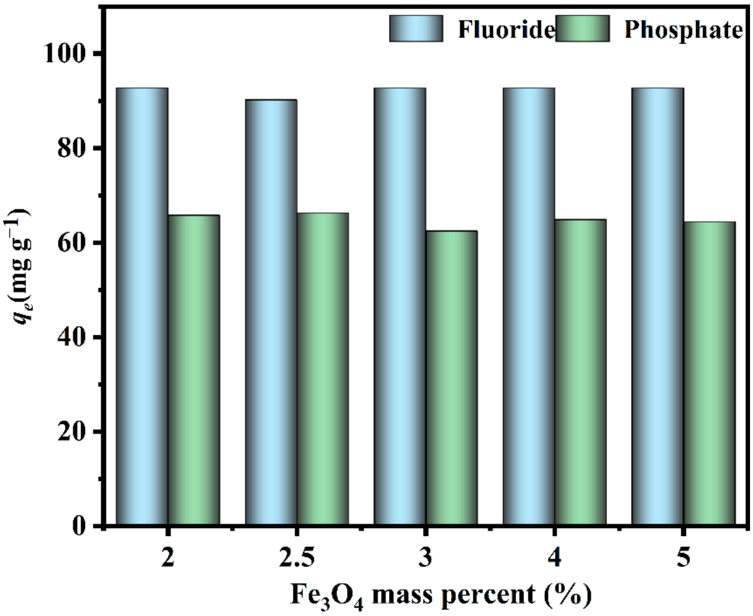

Fig. S1 Effect of Fe_3O_4 dosage for fluoride and phosphate removal

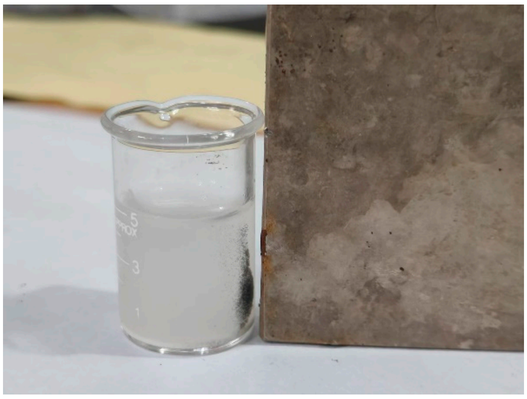

Fig. S2 Digital image of Fe_3O_4@La-Zr-MOFs

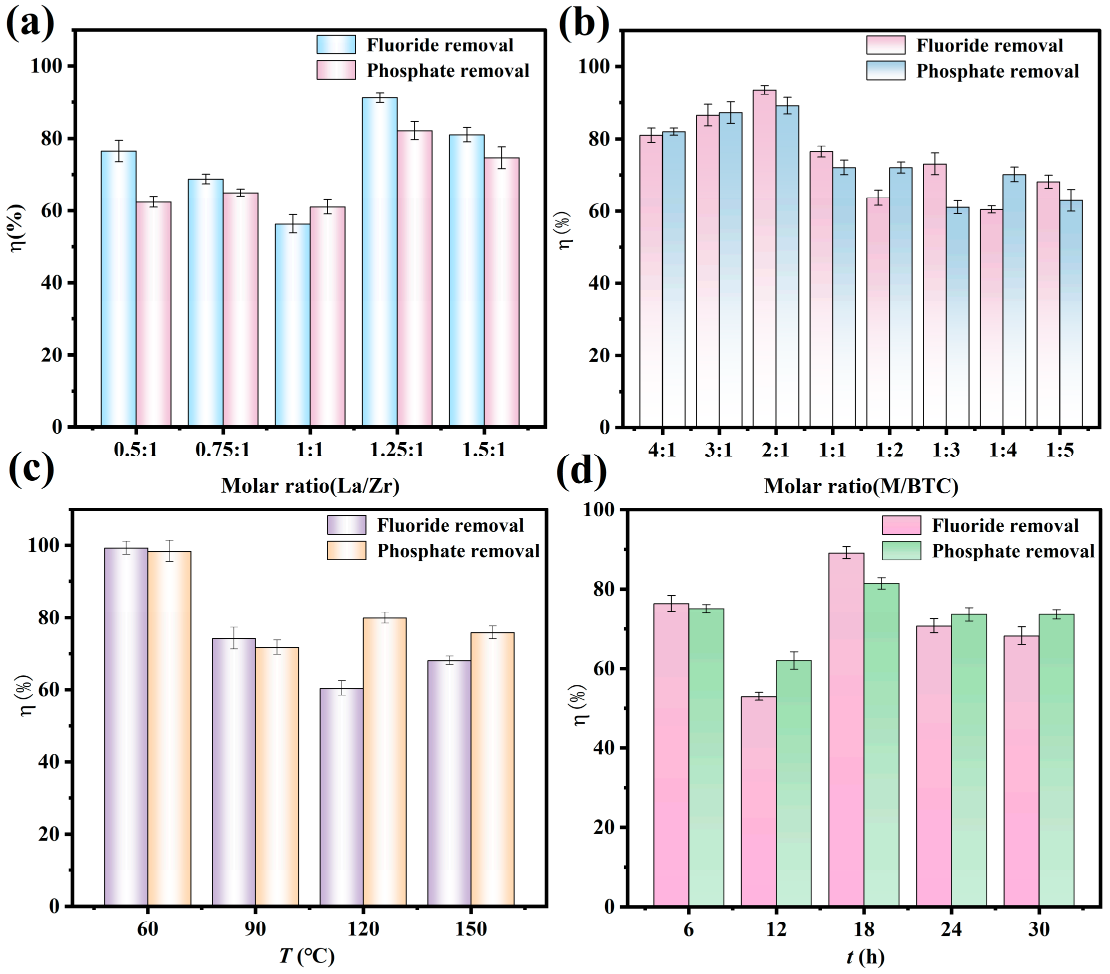

Fig. S3 Effect of (a)Molar ratio (La/Zr), (b) Molar ratio(M/BTC), (c) reaction temperature, (d) reaction time

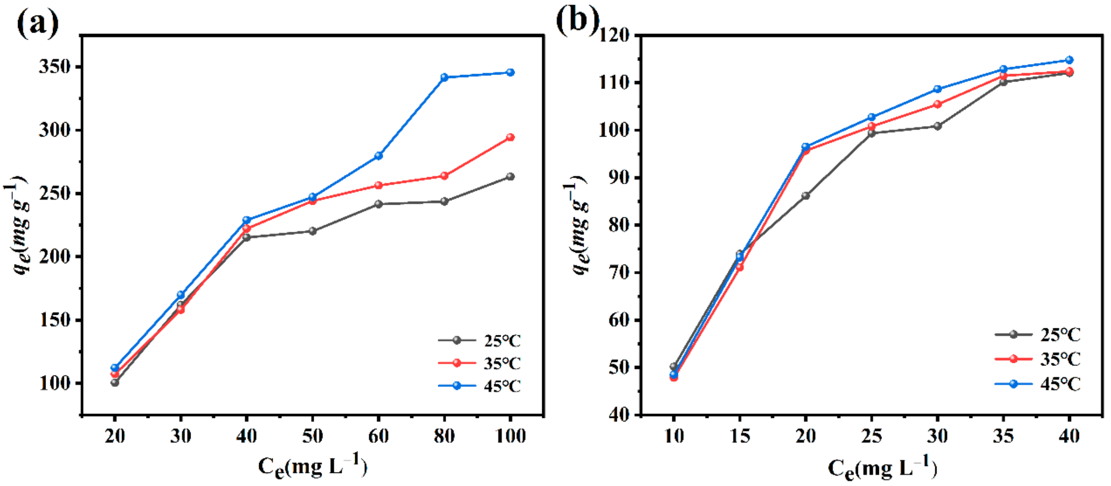

Fig. S4 Adsorption thermodynamics curves for (a) fluoride and (b) phosphate

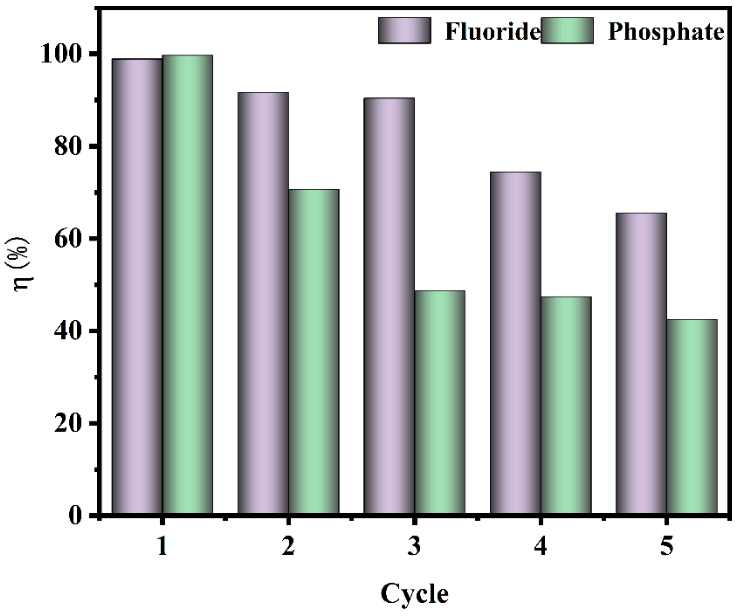

Fig. S5 Regeneration performance of Fe_3O_4@Zr-La-MOFs after fluoride and phosphate removal

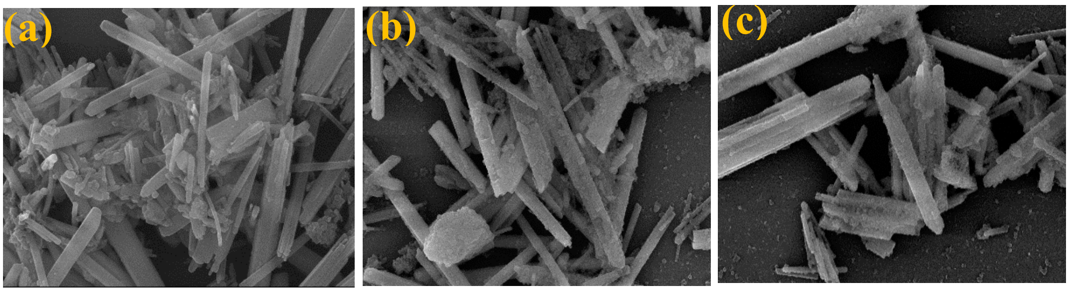

Fig. S6 SEM images of (a) before adsorption (a) after adsorption fluoride (a) after adsorption phosphate

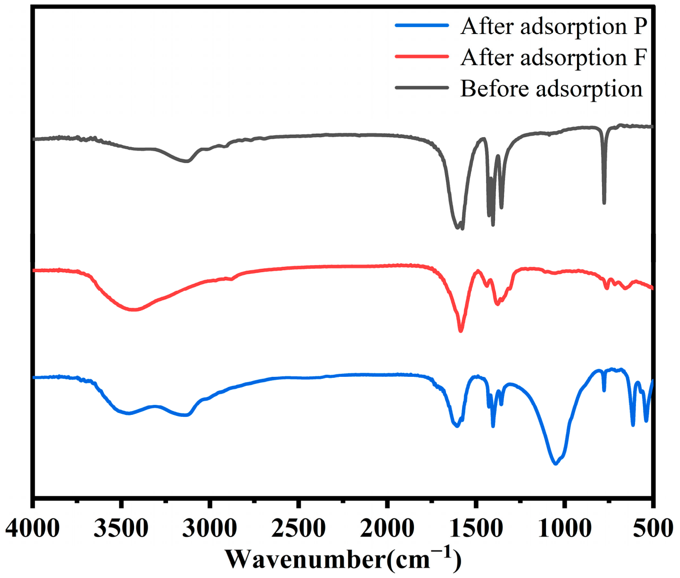

Fig. S7 Fe_3O_4@La-Zr-MOFs FTIR spectra

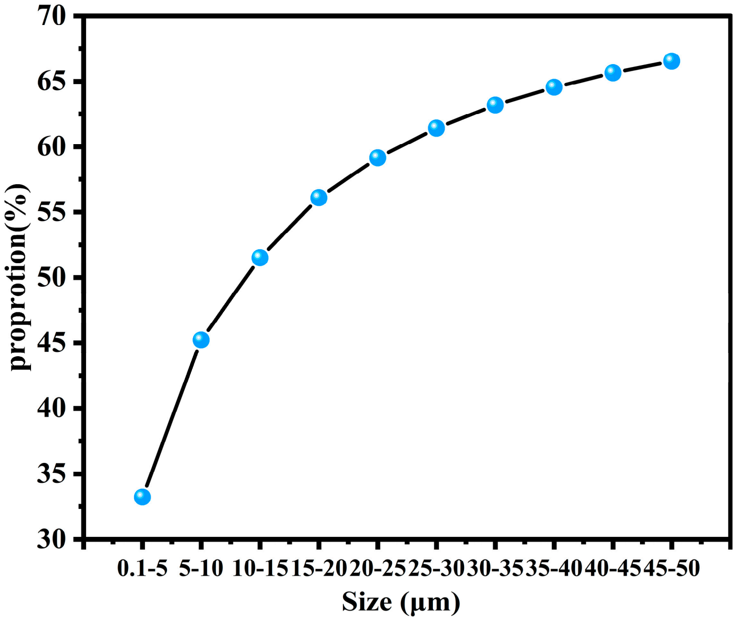

Fig. S8 The particle size distribution of Fe_3O_4@La-Zr-MOFs

Table S1 intra-particle diffusion model fitting parameters (phosphate removal)

**Pollutants**	**Concentration (mg L^{-1})**	**Equation**	R^2
**Phosphate**	10	y=24.9924+6.6782x	0.9093
		y=77.0331+0.8982x	0.7303
		y=89.2689+0.0289x	1
	15	y=42.0536+4.8602x	0.7928
		y=80.5281+0.7484x	0.9014
		y=93.0472+0.0320x	0.9635
	20	y=36.5037+6.1273x	0.9301
		y=73.8469+1.7468x	0.9417
		y=99.5169+0.0289x	1

Table S2 intra-particle diffusion model fitting parameters (fluoride removal)

**Pollutants**	**Concentration (mg L^{-1})**	**Equation**	R^2
**Fluoride**	20	y=170.762+1.7547x	0.8603
		y=181.287+0.1975x	0.8904
		y=184.796+0.0834x	1

		y=192.192+1.4440x	0.7816
	25	y=199.426+0.2964x	0.8655
		y=204.977+0.0379x	1
		y=183.271+2.2085x	0.8081
	30	y=198.052+0.0218x	0.8791
		y=198.053+0.0379x	1

Table S3 The fitting parameters of Langmuir and Freundlich models

Pollutants	T(°C)	Langmuir				Freundlich		
		q_{m}(mg g^{-1})	K_L(L mg^{-1})	R^2	R_L	K_f(L g^{-1})	n	R^2
Fluoride	25	263.85	0.0338	0.9441	0.4251	148.08	6.7521	0.7766
	30	297.61	0.0396	0.9517	0.3869	154.73	6.3532	0.8226
	45	398.40	0.0562	0.9600	0.3078	148.95	4.7123	0.9354
Phosphate	25	156.25	0.0698	0.9280	0.1253	83.69	10.1574	0.9811
	35	189.75	0.0685	0.9224	0.1273	88.42	9.9522	0.8585
	45	197.23	0.0692	0.8988	0.1262	84.13	7.9579	0.7579

Table S4 Thermodynamic parameters of phosphate and fluoride adsorption on Fe_3O_4@La-Zr-MOFs at different temperatures

Pollutants	T (K)	ΔG^o (kJ·mol^{-1})	ΔH^o (kJ·mol^{-1})	ΔS^o (J·mol^{-1}·K^{-1})
Phosphate	298	−4.6371	15.6336	68.0226
	308	−5.3173		
	318	−5.9975		
Fluoride	298	−3.1208	13.1404	54.5681
	308	−3.6665		
	318	−4.2122		

Table S5 Comparison of sorption capacity between Fe_3O_4@Zr-La-MOFs and other adsorbents

Adsorbents	Reaction conditions	Defluoridation		Dephosphorization		References
		pH	q_m (mg g^{-1})	pH	q_m (mg g^{-1})	

Fe_3O_4@La–Ce	C_{F^-}=10-80 mg L^{-1}, C_p=5-60 mg L^{-1}, t=12 h, m/V=0.5 g L^{-1}	4	60.3	3	53.2	[70]
Fe_3O_4@n-HAp Alg composite	C_{F^-}=8-14 mg L^{-1}, t=0.5 h, m/V=2 g L^{-1}	3	4.05	-	-	[71]
$Fe_3O_4/Al_2O_3/ZrO_2$ ternary oxide	C_{F^-} =10-140 mg L^{-1}, t=12 h, C_p=10-140 mg L^{-1}, t=8 h, m/$V_{(F^-)}$=20 g L^{-1}, m/$V_{p)}$=4 g L^{-1}	4	8.97	5	33.33	[72]
Zr-PEI@Fe_3O_4	C_p=5-50 mg L^{-1}, t=8 h, m/V=1 g L^{-1}	-	-	2	32.2	[73]
Fe_3O_4-NPs	C_{F^-}=5-25 mg L^{-1}, t=0.75 h, m/V=0.02 g L^{-1}	6.0	19.44	-	-	[74]
La@MgAl	C_{F^-}=10-200 mg L^{-1}, C_p=10-200 mg L^{-1}, t=24 h, m/V=0.4 g L^{-1}	6.0	51.03	6.0	101.59	[53]
Ce-La bimetal oxides	C_{F^-}=10 mg L^{-1}, C_p=30 mg L^{-1}, t=12 h, m/V=0.5 g L^{-1}	4.0	59.14	4.0	19.25	[75]
La-Zr-D201	C_p=20 mg L^{-1}, t=24 h, m/V=0.5 g L^{-1}			6	61.31	[76]
Fe_3O_4@mSiO_2@mLDH	C_{F^-}=5-30 mg L^{-1}, C_p=10-50 mg L^{-1}, t=24 h, m/V=1 g L^{-1}	5	57.07	5	28.51	[77]
Ce-UiO-66 MOF	CF-=25 mg L^{-1}, t=1 h, m/V=0.4 g L^{-1}	3	66.1	-	-	[78]
Fe-La composite	C_{F^-}=5-20 mg L^{-1}, C_p=50-100 mg L^{-1}, t=1 h, m/V=1 g L^{-1}	3.8 – 7.1	27.41	3.8 – 7.1	89.41	[20]
Ce-La-MOFs	C_{F^-}=10-50 mg L^{-1}, t=12 h, m/V=0.2 g L^{-1}, pH=3	3	138.64			[33]
Ce-H_3TATAB-MOFs	C_{F^-}=10-50 mg L–1, t=12 h, m/V=0.2 g L–1, pH=4	4	129.7			[79]
Fe_3O_4@ Zr-La-MOFs	C_{F^-}=20-100 mg L^{-1}, C_p=10-40 mg L^{-1}, t=12 h, m/V=0.2 g L^{-1}	3	398.40	3	197.23	This study

Table S6 Wastewater quality parameters with and without Fe_3O_4@La-Zr-MOFs

Parameters	Fluoride wastewater		Phosphate wastewater	
	before	after	before	after
Fluoride concentration (mg L^{-1})	11.48	0.52		

Phosphate concentration (mg L^{-1})			14.71	0.45
pH	3.23	3.64	3.21	3.99
NH_3–N (mg L^{-1})	4.44	2.21	3.56	3.06
COD (mg L^{-1})	55	29	102	68
Cl^- (mg L^{-1})	140.47	87.15	142.39	87.56
Na^+ (mg L^{-1})	92.14	91.71	83.43	81.11
HCO_3^- (mg L^{-1})	94.61	60.43	37.82	11.56
SO_4^{2-} (mg L^{-1})	113.57	77.03	115.33	73.84
NO_3^- (mg L^{-1})	58.37	54.03	54.05	50.69
